# Therapeutic-Dose Anticoagulation in Hospitalized Patients With COVID-19: Are We Getting Closer to the Truth?∗

**DOI:** 10.1016/j.jacadv.2023.100779

**Published:** 2024-01-26

**Authors:** David D. Berg

**Affiliations:** TIMI Study Group, Cardiovascular Division, Brigham and Women’s Hospital, Harvard Medical School, Boston, Massachusetts, USA

**Keywords:** anticoagulation, clinical trials, COVID-19, thromboprophylaxis

**Figure:**
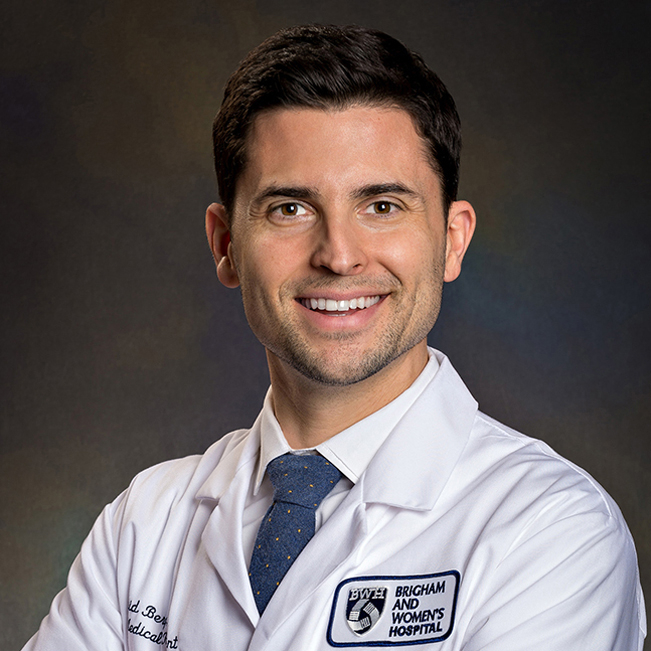

Infection with the SARS-CoV-2 virus carries an elevated risk of thrombosis compared to other viral respiratory infections.^1^ In addition to “macrovascular” thrombotic complications such as venous thromboembolism, early autopsy studies of patients who died from COVID-19-associated respiratory failure demonstrated severe pulmonary vascular endothelialitis and widespread “microvascular” thrombosis.^2^ These early observations fueled the hypothesis that “thromboinflammation” may be a central mechanism driving disease progression in COVID-19, which motivated the design and execution of multiple clinical trials testing anticoagulation strategies in this patient population.

One of the largest such trials was the multiplatform randomized controlled trial (mpRCT), which was a collaboration between 3 independent international trial platforms: ATTACC (Antithrombotic Therapy To Ameliorate Complications of COVID-19), ACTIV-4a (Accelerating COVID-19 Therapeutic Interventions and Vaccines-4 Antithrombotics Inpatient platform trial), and REMPA-CAP (Randomized, Embedded, Multifactorial Adaptive Platform Trial for Community-Acquired Pneumonia).^3^^,^^4^ The mpRCT main analysis population was stratified a priori by severity of illness, with critically ill (ie, those receiving intensive care unit [ICU]-level organ support) and noncritically ill patients analyzed separately. Patients were randomly allocated to receive either therapeutic-dose anticoagulation with heparin or “usual-care” thromboprophylaxis, which included both intermediate- and low-dose prophylactic regimens. The primary endpoint was the number of days without cardiovascular or respiratory organ support through day 21 among patients who survived to hospital discharge, with patients who died during the index hospitalization being assigned a value of −1.

The primary analyses were performed using a Bayesian framework in a modified intention-to-treat (mITT) population. In these analyses, the mpRCT investigators found that in noncritically ill patients (n = 2,219), the probability that therapeutic-dose anticoagulation increased organ-support free days (OSFD) as compared with usual-care thromboprophylaxis was 98.6% (adjusted proportional OR: 1.27; 95% credible interval [CrI]: 1.03-1.58).^3^ By contrast, in critically ill patients (n = 1,098), the probability that therapeutic-dose anticoagulation increased OSFD as compared with usual-care thromboprophylaxis was only 5.0% (adjusted proportional OR: 0.83; 95% CrI: 0.67-1.03).^4^ In other words, there was a convincing benefit of therapeutic-dose anticoagulation on organ function in noncritically ill patients, but not in critically ill patients, and in fact, there was a suggestion of possible harm in the latter group.

In this issue of *JACC: Advances*, Godoy et al^5^ present the results of 2 secondary analyses from the mpRCT that provide important additional information about these thromboprophylaxis strategies in hospitalized patients with COVID-19, namely a prespecified per-protocol analysis, and an exploratory analysis comparing the effect of therapeutic-dose anticoagulation separately against intermediate- and low-dose thromboprophylaxis.

## Why is a per-protocolanalysis of the multiplatform RCT important?

Randomized trials are generally analyzed according to the intention-to-treat (ITT) principle, which means that patients are analyzed based on their randomized treatment assignment, regardless of the treatment ultimately received. The goal of this approach is to preserve the advantages of randomization, namely that participants in the 2 study arms be similar in all respects (both measured and unmeasured) except for the allocated treatment. Nevertheless, if there is incomplete adherence to the trial protocol, ITT analyses may provide misleading estimates of the effect of a particular intervention when the intervention is used as indicated.^6^ This becomes particularly relevant when there are clear competing risks associated with an intervention (as is the case with anticoagulation), and the clinician must weigh the magnitude of potential risk against the magnitude of potential benefit. Although per-protocol analyses may be biased by informative censoring, their strength is that the effect estimates are not influenced by adherence. Thus, ITT and per-protocol analyses should be considered complementary and are generally viewed as providing boundaries for the true effect estimate.

For the per-protocol analyses of the mpRCT, investigators determined the initial stable dose of anticoagulation used in the first 48 hours after randomization. If the administered dose was not consistent with the randomly assigned dose, or if the dosing information was not available, patients were excluded from the analysis. Notably, by restricting eligibility to the first 48 hours after randomization, the per-protocol analyses did not consider the impact of later crossovers (eg, from usual-care thromboprophylaxis to therapeutic-dose anticoagulation in the setting of new-onset atrial fibrillation), which may have also influenced the apparent treatment effect. Therefore, the reported per-protocol analyses were still fairly conservative.

Using this approach, the investigators found that in noncritically ill patients (n = 1,761; 78.9% of mITT population), the probability that therapeutic-dose anticoagulation increased OSFD as compared with usual-care thromboprophylaxis was 99.3% (adjusted OR, 1.36; 95% CrI: 1.07-1.74). The magnitude of the effect estimate was therefore greater in the per-protocol analyses than in the mITT analyses, and based on a modeled absolute increase of 5.1% in the likelihood of surviving to hospital discharge without organ support with therapeutic-dose anticoagulation, the number needed to treat based on the per-protocol analyses was only 20 patients (as compared to 25 in the mITT analyses). The investigators did not model the difference in major bleeding between treatment arms, but the event rates were generally quite low (2.2% vs 1.0% with therapeutic-dose anticoagulation and usual care thromboprophylaxis, respectively).

In addition, the per-protocol analyses suggested that the probability that therapeutic-dose anticoagulation improved overall survival to hospital discharge was 93.7% (adjusted OR: 1.35; 95% CrI: 0.91-2.00). This finding lends additional support to the pattern of lower mortality with therapeutic-dose anticoagulation that has been seen in multiple other trials testing this strategy in noncritically ill patients.^7^, ^8^, ^9^

Among critically ill patients (n = 857; 77.7% of mITT population) enrolled in the mpRCT, the per-protocol analyses again suggested no benefit of therapeutic-dose anticoagulation with respect to reducing the need for organ support or decreasing mortality. However, it is worth acknowledging that the treatment effect on these 2 endpoints (OSFD and in-hospital mortality) was even more neutral in the per-protocol analyses, suggesting that any hint of harm of therapeutic-dose anticoagulation with respect to need for organ support or death in critically ill patients in the mITT analyses may have been spurious.

## What did we learn about intermediate dose thromboprophylaxis?

Recognizing the increased risk of thrombosis in COVID-19, many clinicians adopted a strategy of intermediate-dose thromboprophylaxis early in the pandemic, hoping this might strike the right balance between reducing thrombotic complications and minimizing bleeding risk. However, in the INSPIRATION trial, which enrolled critically ill patients with COVID-19, intermediate-dose prophylaxis did not reduce the primary composite outcome of venous or arterial thrombosis, treatment with extracorporeal membrane oxygenation, or mortality within 30 days compared to standard-dose prophylaxis (OR: 1.06; 95% CI: 0.76-1.48).^10^ This entirely neutral result largely shifted clinical practice away from this strategy. More recently, a multinational, adaptive platform trial conducted in noncritically ill COVID-19 patients, suggested a possible benefit of intermediate-dose prophylaxis but not of therapeutic-dose anticoagulation compared to standard-dose prophylaxis, though the number of patients receiving therapeutic-dose anticoagulation was very small (n = 50).^11^

Against this backdrop, the mpRCT investigators performed an exploratory analysis comparing the effect of therapeutic-dose anticoagulation separately against intermediate- and low-dose thromboprophylaxis, both of which were permitted strategies within the usual-care thromboprophylaxis arm. Notably, there were important imbalances in the characteristics of patients who received intermediate- vs low-dose thromboprophylaxis with respect to illness severity and concomitant therapies, precluding a fully unconfounded comparison of the varying intensities of anticoagulation despite attempts to control for these baseline differences. Nevertheless, therapeutic-dose anticoagulation had a high probability of improving OSFDs relative to both intermediate- and low-dose thromboprophylaxis (99.8% and 94.6%, respectively), lending additional support to the prevailing notion that therapeutic-dose anticoagulation is superior to intermediate-dose prophylaxis.

## What is the optimal thromboprophylaxis strategy in hospitalized patients with COVID-19?

The mixed results of randomized trials testing anticoagulation in hospitalized COVID-19 patients have led to uncertainty about the optimal thromboprophylaxis strategy. Differences in the study designs and study endpoints, heterogeneity in the analytic approaches (Bayesian vs frequentist, ITT vs per-protocol, etc), and apparent treatment effect modification by illness severity have all contributed to a complicated picture. Nevertheless, as the evidence base from randomized trials has gradually matured, that picture has slowly come into clearer focus. When viewed collectively, the results of the mpRCT (both mITT and per-protocol analyses) and other trials evaluating patients who are hospitalized but not critically ill from COVID-19^7^, ^8^, ^9^ appear to suggest that therapeutic-dose anticoagulation decreases the risk of progression to respiratory failure as well as the risk of dying, albeit modestly. By contrast, therapeutic-dose anticoagulation does not appear to decrease the need for organ support or risk of dying once patients have already progressed to the point of critical illness. On the other hand, therapeutic-dose anticoagulation does appear to decrease the risk of venous thromboembolism in critically ill patients,^12^ which is the group that is at particularly high risk for “macrovascular” thromboses. Thus, there is rationale for using therapeutic-dose anticoagulation in all hospitalized COVID-19 patients, but the specific anticipated benefits likely vary based on illness severity and may be most clinically meaningful in noncritically ill patients. Of course, any potential benefit of therapeutic-dose anticoagulation must be weighed against the competing risk of major bleeding, and more work is needed to define which individuals have the optimal risk-benefit profile.

## Funding support and author disclosures

Dr Berg has received research grant support to his institution from 10.13039/100021316AstraZeneca and 10.13039/100004319Pfizer; consulting fees from AstraZeneca, Mobility Bio, Inc, and Youngene Therapeutics; honoraria from the Medical Education Speakers Network (MESN) and USV Private Limited; participates on clinical endpoint committees for studies sponsored by Kowa Pharmaceuticals, Tosoh Bioscience, and Beckman Coulter; and is a member of the TIMI Study Group, which has received institutional research grant support through Brigham and Women’s Hospital from Abbott, 10.13039/100020297Abiomed, Inc, Amgen, Anthos Therapeutics, 10.13039/100020305ARCA Biopharma, Inc, 10.13039/100021316AstraZeneca, Boehringer Ingelheim, Daiichi-Sankyo, Ionis Pharmaceuticals, Inc, Janssen Research and Development, LLC, MedImmune, Merck, Novartis, Pfizer, Regeneron Pharmaceuticals, Inc, Roche, Saghmos Therapeutics, Inc, Siemens Healthcare Diagnostics, Inc, Softcell Medical Limited, The Medicines Company, Verve Therapeutics, Inc, and Zora Biosciences.
